# Biosorption of lead, copper and cadmium using the extracellular polysaccharides (EPS) of *Bacillus* sp., from solar salterns

**DOI:** 10.1007/s13205-016-0498-3

**Published:** 2016-09-07

**Authors:** Syed Shameer

**Affiliations:** Department of Microbiology, Sri Venkateswara University, Tirupati, 517 502 AP India

**Keywords:** Solar, Salterns, *Bacillus* sp., Extracellular polysaccharides and metal biosorption

## Abstract

Extracellular Polysaccharides (EPS) from both prokaryotes and eukaryotes have a great deal of research interest as they protect the producer from different stresses including antibiotics, ionic stress, desiccation and assist in bio-film formation, pathogenesis, adhesion, etc. In this study haloalkaliphilic *Bacillus* sp., known to cope with osmophilic stress, was selected and screened for EPS production. The EPS were isolated, partially purified and chemical characteristics were documented using liquid FT-IR followed by assessment of heavy metal biosorption (lead, copper and cadmium) using Atomic Absorption Spectroscopy (AAS). The EPS extracted from three isolates *B. licheniformis NSPA5*, *B. cereus NSPA8* and *B. subtilis NSPA13* showed maximum biosorption of Lead followed by Copper and Cadmium. Of the tested isolates, the EPS from isolate *B. cereus NSPA8* showed maximum (90 %) biosorption of the lead.

## Introduction

EPS are polymeric substances excreted by the microorganisms when growing in environment of abundance or after establishing in a suppressing one and play a major role in the formation of bio-films to grow on the substrates in environments where normally others cannot colonise (Sutherland 2001). In some species, EPS play an essential role in imparting antibiotic resistance to the organism by denying permeability to the antibiotics (Costerton et al. 1987).

Depending on the genera these EPS are made of different carbohydrates, proteins and their derivatives as hetero as well as homo-polymers, which make them potential contender for multiple field applications. EPS are being actively employed in food, bioremediation and pharmaceutical sectors owing to their gelling nature which allows them to be used as preservative, viscosifying agent, flavouring agent and form super-absorbing gels, biosorption agents etc., (Sutherland 1998; Challouf et al. 2011). It is well established that these EPS have large surface area of interaction particularly with cations (metals), which comes handy during surviving in diverse contaminated areas and the same is applicable in remediation of heavy metal contamination (Pal and Paul 2008).

In this study, a moderately haloalkaliphilic bacteria of the genus *Bacillus* was studied from artificial hyper saline habitats i.e., solar salterns, which are wide spread in distribution and primarily present in the tropical and sub-tropical regions constructed by humans near coastal regions for the purpose of edible salt production. As the current knowledge regarding potential applications of microbes from artificial hyper saline environments and their applicability in metal detoxification is very limited, this study was focused to evaluate the metal biosorption ability of EPS extracted from three different types of haloalkaliphilicbacteria.

## Materials and methods

### Isolation, selection and characterisation of *Bacillus* sp., from solar salterns

For the isolation of the *Bacillus* sp., 1.0 g of soil sample was collected from solar salterns by employing standard method of soil sampling (Carter 1993) and was inoculated into 100 ml of the modified nutrient medium with 7 % NaCl and the final pH was adjusted to 8.2. After inoculation, flasks were incubated on orbital shaker at 130 rpm/min with regular monitoring of the turbidity of the media at 37 °C. After 48–72 h of growth, loop full of culture was spread plated**/**pour plated on the nutrient agar (Agar 1.5 % w/v) plate and incubated at 37 °C for 5 days. Based on the colony characteristics such as form, elevation and margin various discrete and distinct colonies were selected and purified. The selected isolates were screened for standard biochemical reactions to establish preliminary identity of the isolates (Cappuccino et al. 2005). Molecular characterization was carried out according to the protocols of Sambrook et al. (1989), Saiki et al. (1988), Weisburg et al. (1991) and Higgins et al. (1992) for genomic DNA isolation, PCR amplification, Amplicon sequencing and phylogenetic relationship analysis, respectively.

### Screening for extracellular polysaccharides (EPS) production

EPS production was screened by visual and by microscopic observation. The colonies were picked from pure culture plates of three potential isolates and spread plated using a disposable L-rod and incubated at 37 °C for 48 h. Mucoidness of colonies was determined by visual appearance and the ropiness of colonies in liquid broth was determined by testing with a sterile inoculation needle. Further EPS production was also confirmed by scanning electron microscopy according to Bulla (1970) and Kaláb et al. (2008) where the isolate’s EPS layers were picked up and transferred to a particle free graphite cover slip and dried at 35 °C and processed for SEM imaging.

### Production and extraction of extracellular polysaccharides

All the three isolates were inoculated into EPS production medium containing Glucose 10.0 g/L, Yeast extract 3.0 g/L, Malt extract 3.0 g/L, Peptone 5.0 g/L, MgSO_4_·7H_2_O 1.0 g/L, KH_2_PO_4_0.3 g/L, vitamin B1 0.001 g/Land pH 7.0 (Banerjee et al. 2009) and incubated in rotatory shaking incubator at 180 rpm for 96 h at 40 °C. After 96 h of incubation the cultures were treated with 10 psi steam for 20 min to loosen the attached polymer, centrifuged at 8000 rpm and supernatant was collected. To the supernatant, chilled Iso-proponal was added in equal volume. This mixture was agitated for few minutes and left overnight at 4 °C (Brown and Lester 1980; Donota et al. 2012). The precipitated EPS were separated by centrifugation at 7000 rpm for 20 min. The supernatant was dried in an oven at 60 °C until it reaches constant weight. The precipitate was used for protein analysis and dried supernatant was used for total carbohydrate and reducing sugars estimation.

### Dry weight of the extracellular polysaccharides

Extracted EPS were dried in a pre weighed density bottles at 60 °C till it attains constant weight. Dry weight of polymer was calculated in relation to the volume of supernatant used for extraction (Ohno et al. 2001).

$${\text{Weight of dry polymer}} = \varvec{B} - \varvec{A}$$

***A*** weight of empty density bottle, ***B*** weight of bottle with dry polymer

### Characterisation of extracellular polysaccharides

#### Characterisation of physical properties of EPS

The physical properties of EPS such as colour and texture were analysed by macroscopic and Electron microscopic observation. The dried EPS layer was placed on the particle free carbon tape and attached to the metallic studs and placed in the Gold sputtering chamber to coat the particles to create electron dense and sparse regions. The difference in the gold coated particles gives dark and brighter regions creating an electron image (Sutton et al. 1994).

#### Chemical composition of extracellular polysaccharides

The extracted EPS were subjected to total carbohydrate and reducing sugar estimation as described by Dubois et al. (1956), where 2.0 ml of EPS solution was taken into a test tube and 0.05 of 80 % phenol is added followed by rapid addition of 5.0 ml concentrated sulphuric acid. The tubes were allowed to stand for 10 min then, shaken well and placed in water bath at 30 °C for 20 min. The absorbance was measured at 490 nm and calculated against a standard of glucose treated similarly.

The reducing sugars were estimated following DNS method, where 1.0 ml of EPS was mixed with 3.0 ml of DNS reagent and kept in a boiling water bath for 5.0 min and allowed to cool and absorbance was measured at 540 nm, concentration was calculated using standard curve.

The EPS were subjected to protein estimation as per Lowry et al. (1951), where to 1.0 ml of EPS solution 4.5 ml of alkaline copper sulphate reagent is added and incubated at room temperature for 10 min. Then 0.5 ml of Folin-Phenol reagent is added and incubated for further 30 min and absorbance was measured at 660 nm, protein concentration was calculated against a standard curve.

#### Structural elucidation of the extracellular polysaccharides

Structural elucidation of the EPS was done using Fourier Transform Infrared Spectroscopy (FT-IR, from Perkin Elmer, Germany). For FT-IR analysis, 2.0 mg of extracted EPS was grounded with 200 mg of KBr and then pressed in a mould. The absorption spectrum was recorded in the frequency range of 4000–400 cm^−1^ to analyse different functional groups present in the EPS. Thus, obtained pellets were analysed for different functional groups to asses’ structural characters (Lijour et al. 1994; Verhoef et al. 2005; Denkhaus et al. 2007).

### Metal biosorption by extracellular polysaccharides

The metal biosorption potential of the EPS against heavy metals Lead, Copper and Cadmium was studied by adding the whole EPS at 1.0 mg/100 ml of the test metal solution having 1000 ppm of respective metal at 37 °C under constant stirring of 250 rpm for 24 h. After incubation, the EPS were separated by centrifugation at 10,000 rpm and the metal biosorption was measured using Atomic Absorption Spectroscopy (AAS) (Bass et al. 2001). The metal biosorption potential is deducted by calculating as follows (Volesky and Holan 1995). Metal removal (q), from the solution was expressed as mg metal removed/g dry weight^−1^, which was calculated using the following formula

$$q \, \left( {{\text{mg g}}^{ - 1} } \right) = V\left( {C_{i} {-}C_{f} } \right){\text{ m}}^{ - 1}$$

where **V** is the sample volume (l), **C**_**i**_ and **C**_**f**_ are the initial and final metal concentrations (mg/l), respectively, **m** is the amount (g) of dry biomass.

## Results

### Extracellular Polysaccharides production and applications

#### Isolation, selection and characterisation of *Bacillus* sp., from solar salterns

**Fig. 1:**
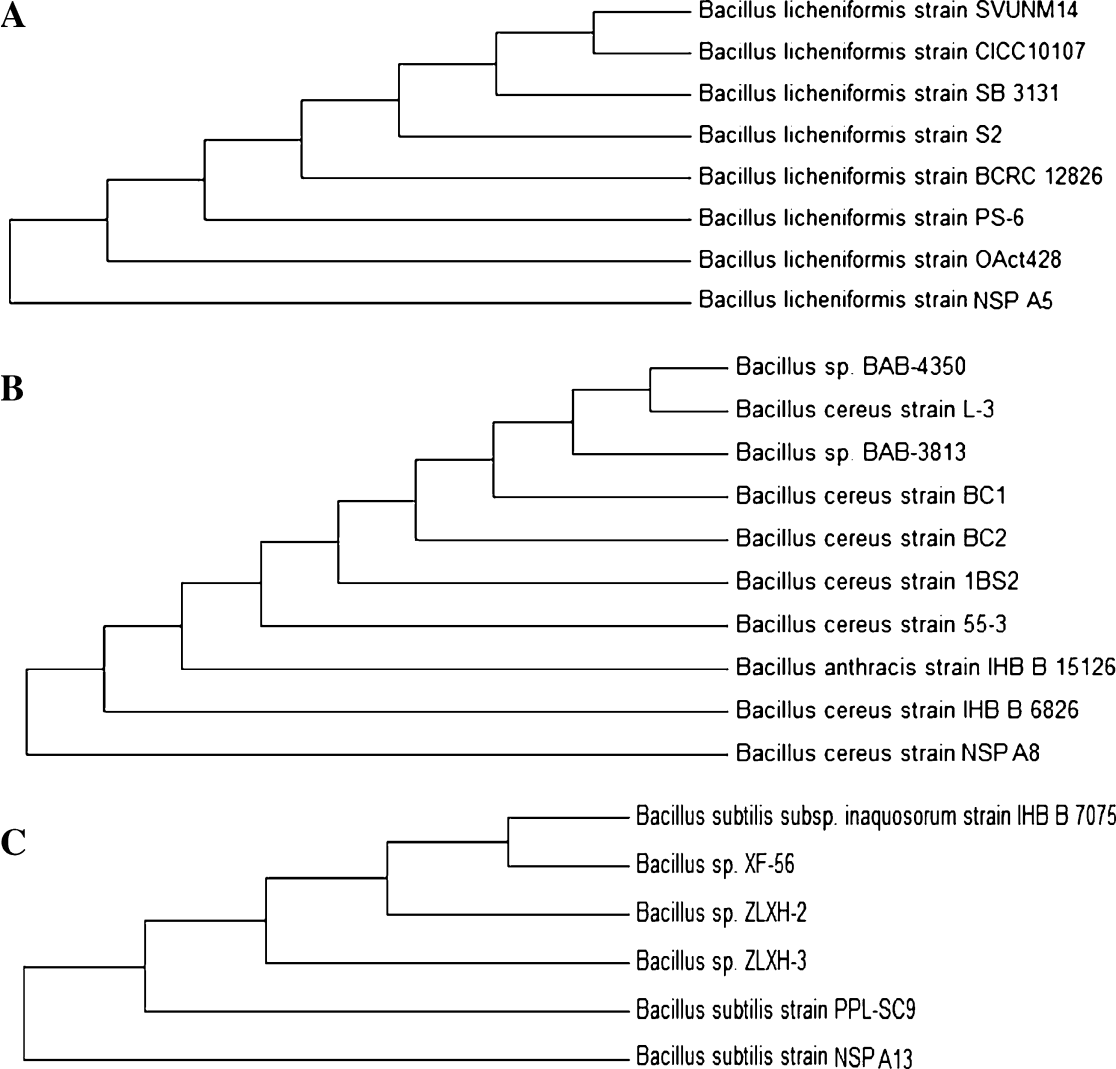
Phylogenetic analysis of the isolates based on 16S rDNA sequence analysis. **a** Phylogenetic tree of isolate NSPA5; **b** Phylogenetic tree of isolate NSPA8; **c** phylogenetic tree of isolate NSPA13

A total of 14 bacterial isolates were initially isolated from solar salterns on modified nutrient agar medium and the isolates were selected on the basis of cultural characteristics such as colony size, colour, form, margin and elevation. Based on the Bergey’s manual of systemic bacteriology, those fitting the description of *Bacillus* sp were selected for molecular characterisation (Bergey et al. 1939). The selected isolates were taxonomically classified using phylogenetic analysis. The amplified 16S rDNA gene using polymerase chain reaction resulted in a single discrete band of a 1.5 kb size in agarose gel. This amplified PCR product was BLAST searched against NCBI Genbank and RDP (Ribosomal Database Project) database 11.0. A distance matrix was constructed based on nucleotide sequence homology using kimura-2 parameter and phylogenetic trees were made using neighbour joining method. Based on nucleotide homology and phylogenetic analysis the isolates NSPA5, NSPA8 and NSPA13 showed highest similarity (99.0 %) with *Bacillus licheniformis* (Genbank Accession no AB301011) and nearest homolog was found to be *Bacillus* sp. (Genbank FR823409), *Bacillus cereus st.*GUFBSS253-84 (Genbank. JN315893) 99 %, respectively, and nearest homolog was found to be *Bacillussp*.BP9_4A (Genbank JN644555) and *Bacillus subtilis st.HS*-*116* (Genbank JQ062996) 99 % and nearest homolog was found to be *Bacillus subtilis st.*69 (Genbank JN582031), respectively. The sequences were submitted to Genbank with Accession No: JQ922113, KC686834 and KC686835 for the sequences of NSPA5, NSPA8 and NSPA13respectively (Fig. 1).

### Screening for extracellular polysaccharide (EPS) production

**Fig. 2:**
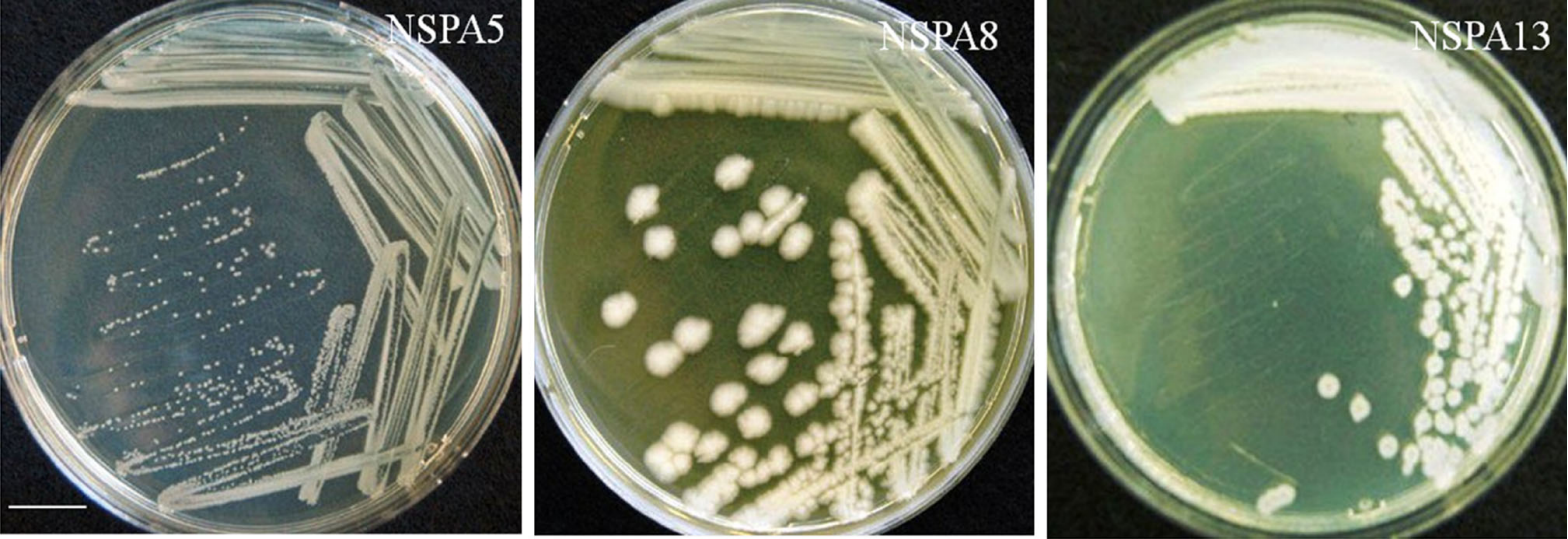
Isolates showing mucoidal growth on modified nutrient agar medium. *Bar* indicates 2.5 cm

**Fig. 3:**
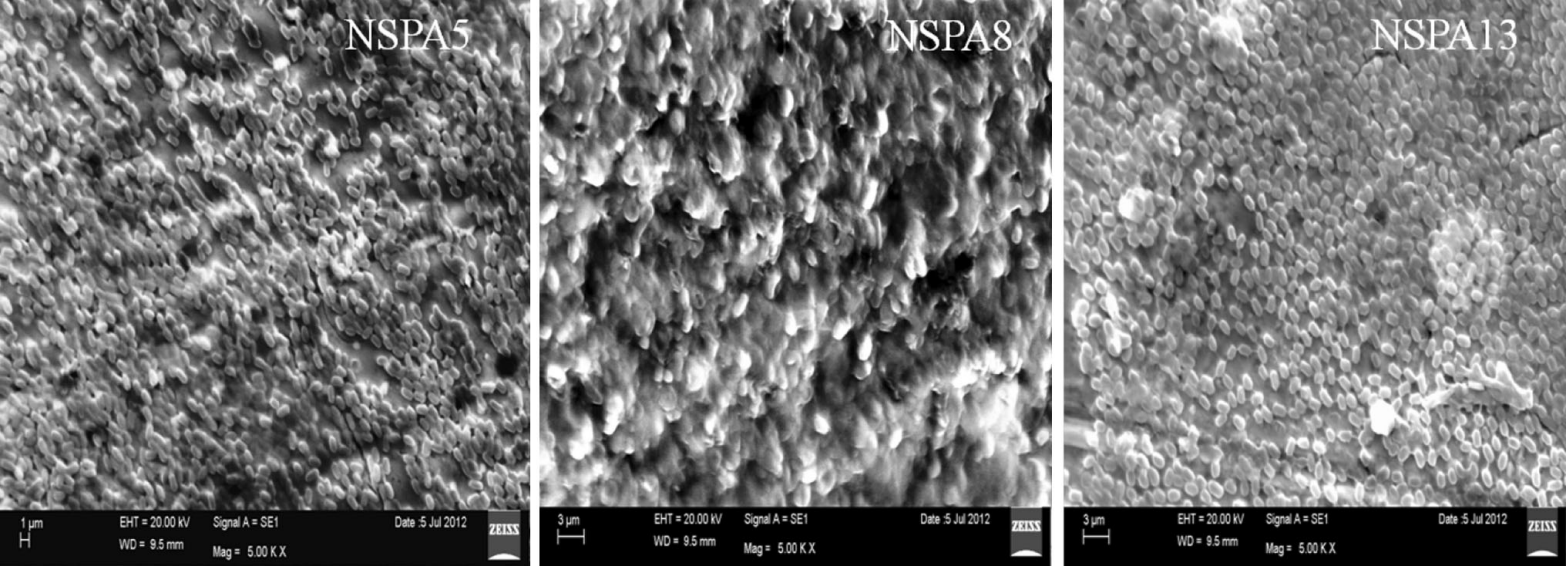
SEM images of Isolates showing extracellular polysaccharides attached to the cells and the cells growing as a bio-film

The isolates *B. licheniformis NSPA5*, *B. cereus NSPA8* and *B. subtilis NSPA13* showed mucoidal growth during their isolation and purification. The bacterial cells were clearly observed as encapsulated by the EPS through capsule staining and gram’s staining observations. Further the SEM images clearly established the presence of extracellular polymer attached to the bacterial cells, and the cells grew as a Bio-film. Images showing the Bio-film formation are presented below (Figs. 2, 3).

### Dry weight of the extracellular polysaccharides

The EPS total dry weight of the isolates *B. licheniformis NSPA5*, *B. cereus NSPA8* and *B. subtilis NSPA13* measured up to around 22.25, 21.2 and 19.3 mg/10 ml, respectively. The amount of EPS produced varied even though the growth medium and condition were identical.

### Characterisation of extracellular polysaccharides

#### Characterisation of physical properties of EPS

**Fig. 4:**
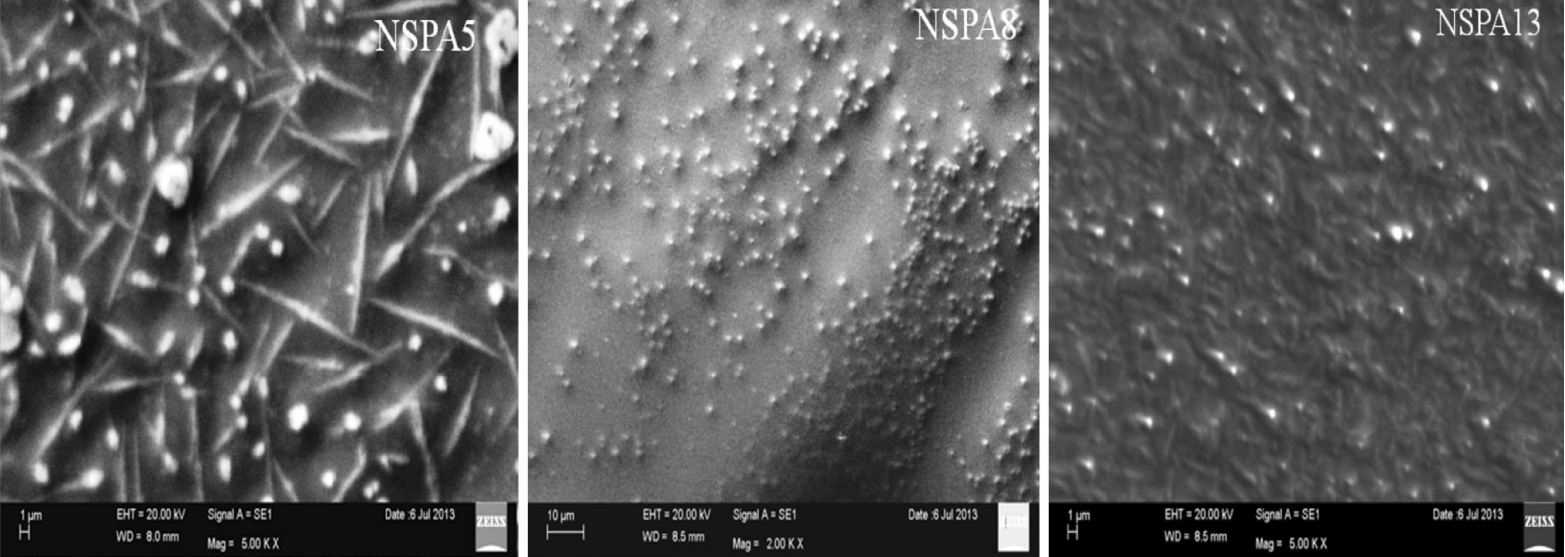
SEM images of dried EPS of isolates after separation by Iso-proponal precipitation

**Fig. 5:**
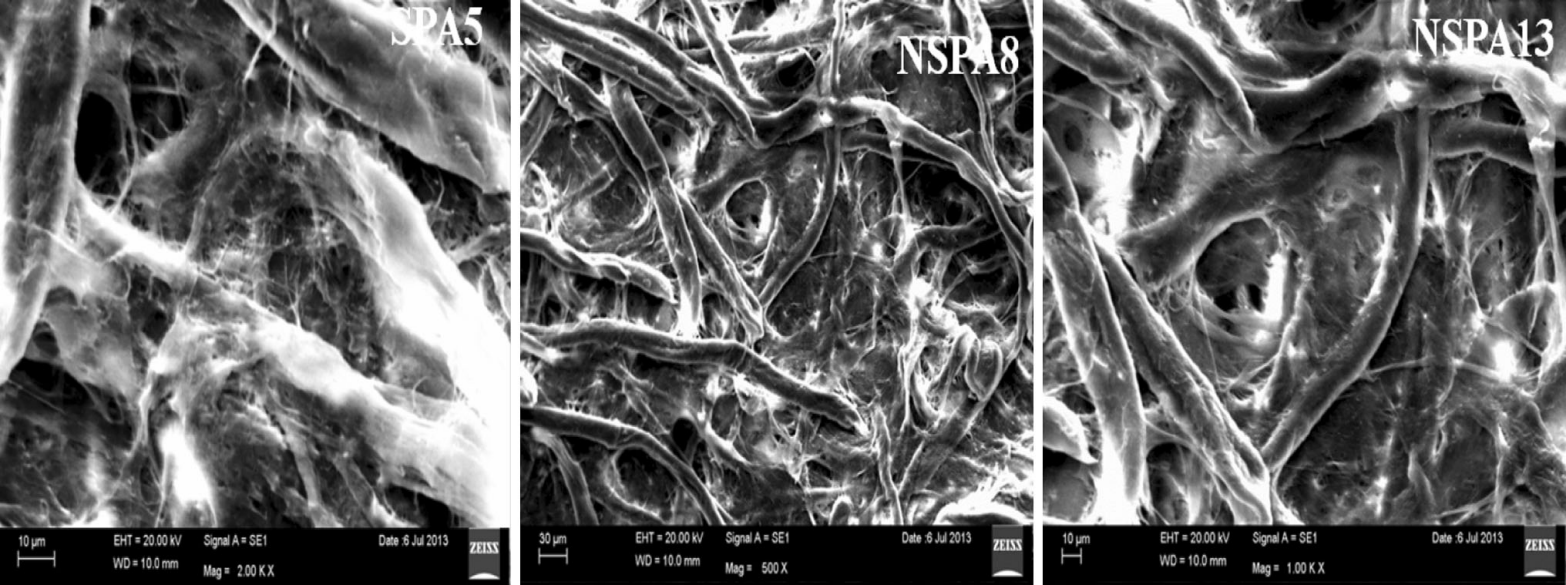
SEM images of mucoidal layers of the isolates showing highly dense polymeric structures

The EPS of the isolates was granular powder in case of *B. licheniformis NSPA5* and *B. subtilis NSPA13* but that of *B. cereus NSPA8* was finely amorphous in nature from naked eye examination. Similar characteristics were established by SEM image analysis of the dried powdered EPS and mucoidal layers of the isolates. SEM imaging elucidates Mucoidal layers as intact relatively long polymeric in nature; while the precipitated EPS is highly fragmented when compared with mucoidal layers. The isolate *B. cereus NSPA8* has very thin polymeric chains than the rest of the isolates i.e., *B. licheniformis NSPA5* and *B. subtilis NSPA13.* It is clearly evident that alcohol treatment almost completely denatured the EPS of all the isolates apparent from the SEM images as shown below (Fig. 4) when compared with the untreated ones (Fig. 5).

#### Chemical composition of EPS

**Fig. 6:**
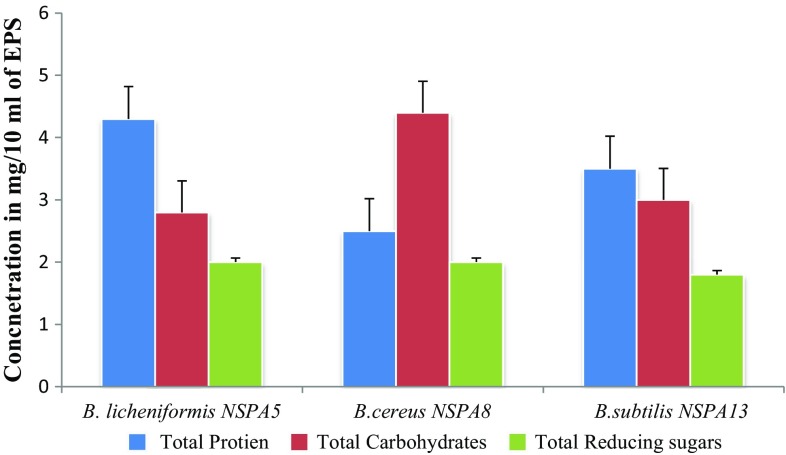
Characteristics of Extracellular polysaccharides from the isolates

The total carbohydrate content in the Extracellular polysaccharide after extraction by precipitation was found to be 2.8, 4.4 and 3 mg/100 mg of the total EPS produced by *B. licheniformis NSPA5*, *B. cereus NSPA8* and *B. subtilis NSPA13,* respectively. Total carbohydrate determination shows isolate *B. cereus NSPA8* has produced maximum and *B. licheniformis NSPA5* the least amount of carbohydrates among the three tested isolates. The same were found to be comprised of reducing sugars which were found to be around 2, 2 and 1.8 mg/100 mg of EPS of *B. licheniformis NSPA5*, *B. cereus NSPA8* and *B. subtilis NSPA13,* respectively. The isolates *B. licheniformis NSPA5* and *B. cereus NSPA8* contained similar amount of reducing sugars in their EPS. The total protein content in the EPS was 4.3, 2.5 and 3.5 mg/100 mg of EPS from *B. licheniformis NSPA5*, *B. cereus NSPA8* and *B. subtilis NSPA13,* respectively; isolate *B. licheniformis NSPA5* produced maximum content in its EPS. The results are presented in Fig. 6.

#### Structural elucidation of the EPS

**Fig. 7:**
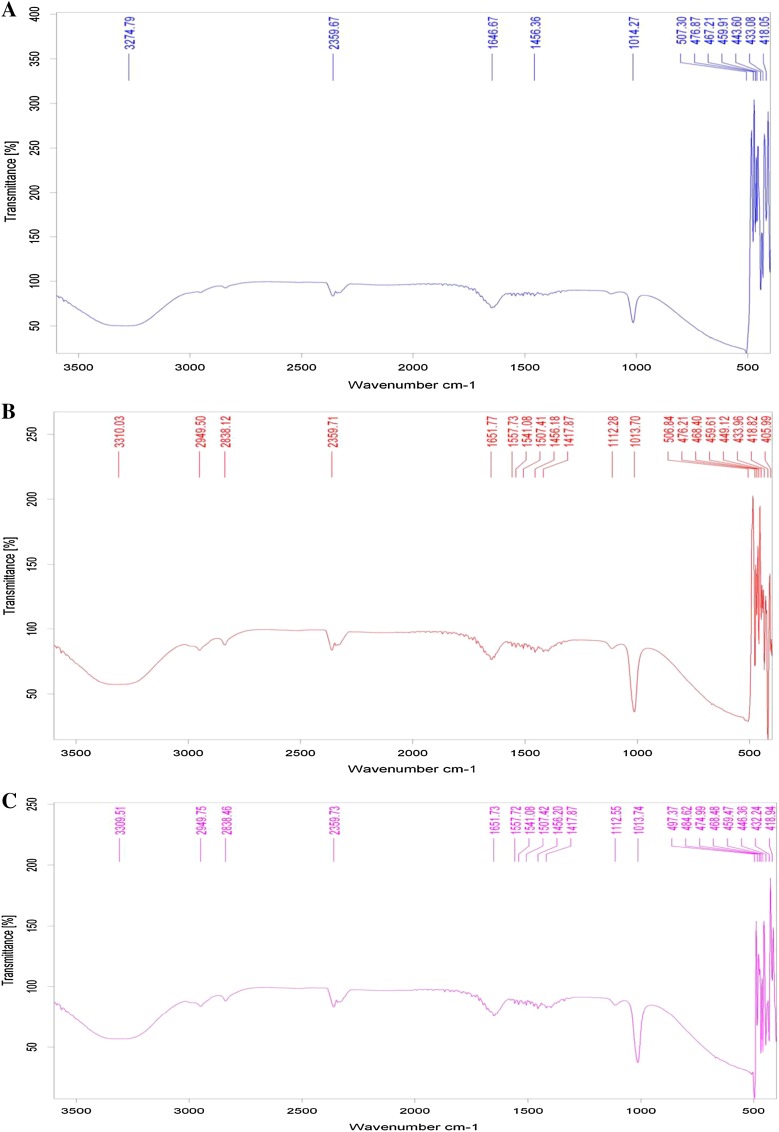
FT-IR spectrum showing characteristic peaks along with finger print Zone of precipitated EPS of isolates. **a** *B. licheniformis NSPA5*; **b** *B. cereus NSPA8*; **c** *B. subtilis NSPA13*

IR spectra of EPS from isolates are presented in Fig. 7. The spectra of three EPS are similar and indicate the presence of the same functional groups mentioned in Table 1. Several intense characteristic bands can be attributed to protein and polysaccharide functional groups. These different functional groups observed agree with results of (Guibaud 2003, 2005) and are in accordance with the EPS biochemical composition.

**Table 1:** Main functional groups observed from IR-spectra of soluble and bound EPS of *B. licheniformis NSPA5*, *B. cereus NSPA8* and *B. subtilis NSPA13*

Wave number (cm^−1^)	Vibration type	Functional type
3400–3300	O–H stretch	Alcohols
2950–2800	C–H stretch	Alkanes
~2850 and ~2750	C–H aldehyde stretch	Aldehydes
3400–2400	O–H stretch	Carboxylic acids
1680–1630	C=O stretch	Amides
1570–1515	N–H bend (1°)	Amides
1550–1490	–NO_2_ (aromatic)	Nitro groups
~1465	CH_2_ bend	Alkanes
1440–1400	O–H bend	Carboxylic acids
1112	C–O bending	Proteins and carbohydrates
1013	C–O bending	Polysaccharides
500–400	S–S stretching < finger print region>	Disulfides

### Metal biosorption by EPS of *B. licheniformis NSPA5*, *B. cereus NSPA8* and *B. subtilis NSPA13* atomic absorption spectroscopy (AAS)

After analysing the treated samples in AAS, the EPS of isolate *B. cereus NSPA8*showed maximum biosorption of the tested metals. The results show all the three isolates were able to adsorb lead at a concentration of 1000 ppm. The metals which expressed lesser inhibition effect in the metal tolerance assay in this case copper and cadmium were the least adsorbed ones; the metal concentration in the bacterial treated medium is reduced to 210.45, 140.2 and 154.65 ppm by the EPS of *B. licheniformis NSPA5*, *B. cereus NSPA8* and *B. subtilis NSPA13,* respectively, in the case of Lead. Copper biosorption was somewhat different as the EPS showed varied biosorption when compared with other two metals; all the three EPS’s showed very distinct abilities as compared with Lead and Cadmium. The EPS of *B. licheniformis NSPA5*, *B. cereus NSPA8* and *B. subtilis NSPA13* reduced the metal concentration to 985.42, 840.5 and 920.48 ppm, respectively, from the original 1000 ppm concentration. In case of copper biosorption all the EPS’s limited themselves to reducing the initial metal concentration to around 942.85, 945.03 and 928.28 ppm by the EPS of *B. licheniformis NSPA5*, *B. cereus NSPA8* and *B. subtilis NSPA13,* respectively, showing uniformity in the copper biosorption ability unlike with Lead and Cadmium.

**Fig. 8:**
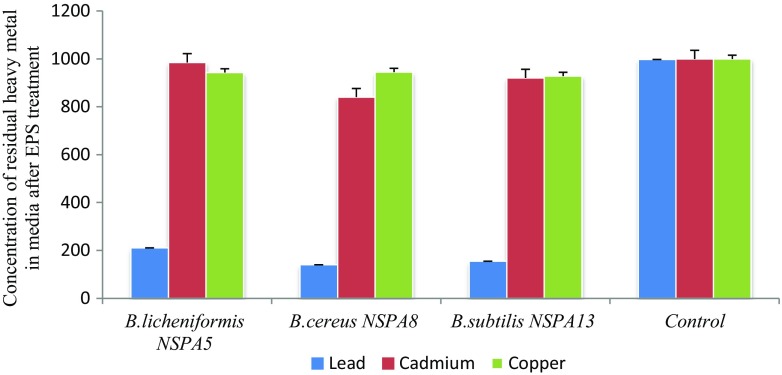
Biosorption of heavy metals by the EPS of the isolates as determined by AAS

The biosorption studies of the selected metal showed that the metal’s toxicity plays crucial role in the biosorption ability, the EPS of *B. cereus NSPA8* showed maximum biosorption of all the three tested metals except copper. Lead was the maximum adsorbed one at almost 90 %. The EPS of *B*. *licheniformis NSPA5* and *B. subtilis NSPA13* also showed maximum biosorption of lead but still less when compared with *B. cereus NSPA8*. The other two metals cadmium and copper were too adsorbed but, negligible when compared with lead. The metal biosorption efficiency of the EPS of the isolates is depicted in the Fig. 8 below as determined from AAS.

## Discussion

In recent years, exopolysaccharides (EPS) produced by bacteria have been employed in diverse fields ranging from food to pharma. So, in this study the EPS produced by the three isolates *B. licheniformis NSPA5*, *B. cereus NSPA8* and *B. subtilis NSPA13* were extracted and its characteristics and metal biosorption were studied. The chemical and dry weight analysis revealed the EPS from the isolates has both carbohydrate and protein components and the quantity produced were more or less in agreement with previous reports (Maria et al. 1996; Shih et al. 2010). The same was contradicted by reports from Mitsuda et al. (1981) and Shih et al. (2010) where EPS were majorly composed of carbohydrates and their derivatives. FT-IR analysis showed the presence of active carboxylic groups and the results were in harmony with reports other workers like Suh et al. (1993) and Ganesh et al. (2004) where Haloalkaliphilic *Bacillus* sp. was studied. The EPS when screened for heavy metal biosorption showed variation in results to that of previous reports (James 1986; Chen et al. 2002; Salehizadeh and Shojaosadati 2003; Yilmaz 2003) when *Bacillus* sp, was studied. The major difference being EPS in this study has significant biosorption of Lead only but above reports indicates it was the least biosorbed. In case of Copper and Cadmium which was higher in their reports, our study showed only minimal biosorption. All the isolate’s EPS biosorbed the tested metals Lead, Cadmium and Copper, but Lead was the most sorbed by all the isolates as determined by AAS at a concentration of 1000 ppm, but previous studies were with lower concentrations at around 100 ppm only. Thus, EPS from haloalkaliphilic *Bacillus* sp. have potential applications in treatment of metal contaminated waters specially lead contamination as proposed by Ha et al. (1991) (Loaëc et al. 1997; Beyenal and Lewandowski 2004; Pal and Paul 2008).
